# Supplementary material for: The causal role of circulating inflammatory markers in osteoporosis: a bidirectional Mendelian randomized study
Source: Front Immunol. 2024 Jul 18;15:1412298. doi: 10.3389/fimmu.2024.1412298 (PMC11291241; doi:10.3389/fimmu.2024.1412298)
Supplement: Supplementary file 1 [file DataSheet_1.zip › supplementary material/OSTEOPOROSIS-CIM/GCST90274806.tsv.gz_leave_one_out.pdf]

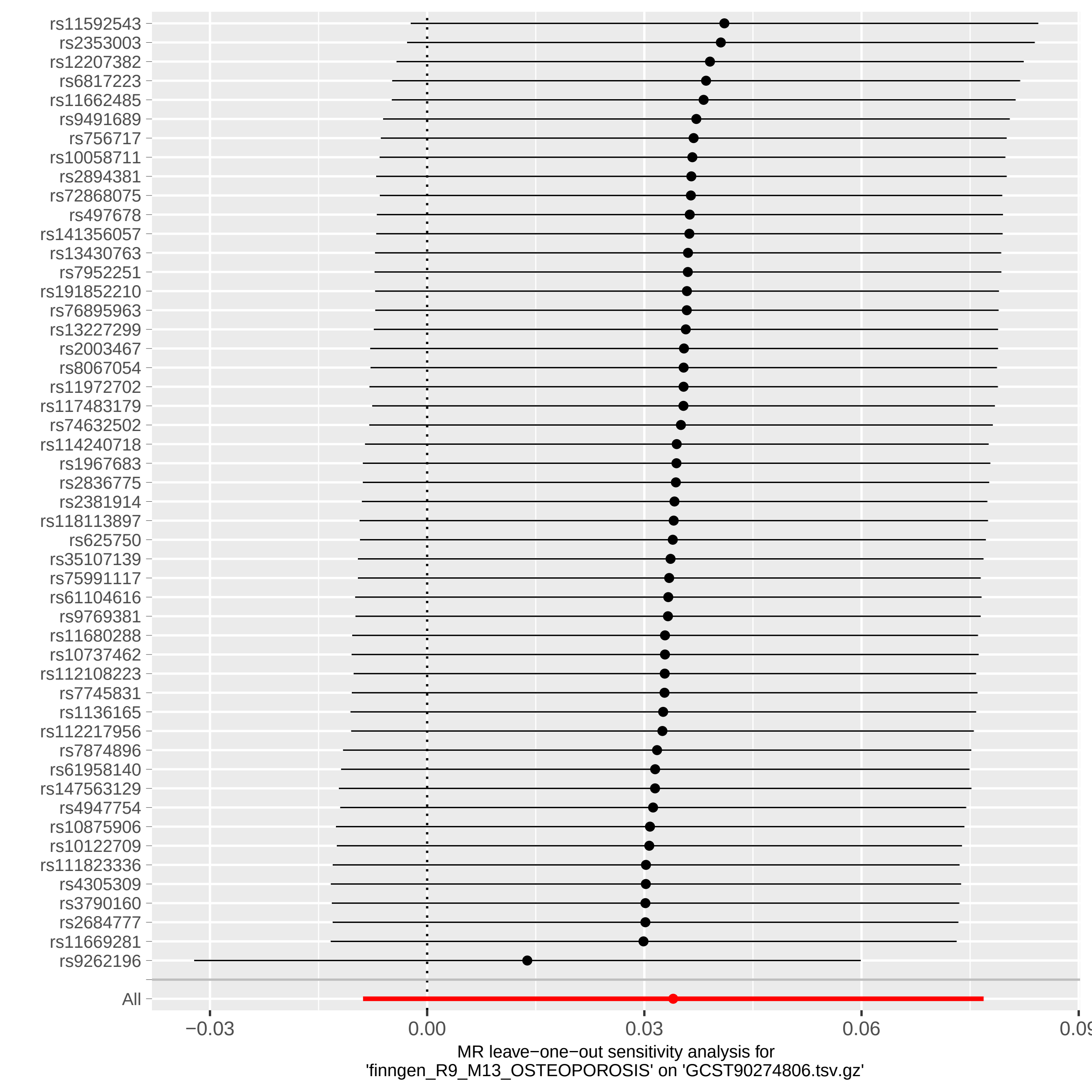

rs11592543
rs2353003
rs12207382
rs6817223
rs11662485
rs9491689
rs756717
rs10058711
rs2894381
rs72868075
rs497678
rs141356057
rs13430763
rs7952251
rs191852210
rs76895963
rs13227299
rs2003467
rs8067054
rs11972702
rs117483179
rs74632502
rs114240718
rs1967683
rs2836775
rs2381914
rs118113897
rs625750
rs35107139
rs75991117
rs61104616
rs9769381
rs11680288
rs10737462
rs112108223
rs7745831
rs1136165
rs112217956
rs7874896
rs61958140
rs147563129
rs4947754
rs10875906
rs10122709
rs111823336
rs4305309
rs3790160
rs2684777
rs11669281
rs9262196
All
−0.03
0.00
0.03
0.06
0.09
MR leave−one−out sensitivity analysis for
'finngen_R9_M13_OSTEOPOROSIS' on 'GCST90274806.tsv.gz'
